# The Impact of Magnetic Resonance Imaging Findings in Predicting Neurological Status Pre- and Post-Treatment of Spinal Dural Arteriovenous Fistulas: A 22-Year Experience in a Neurovascular and Spine Center

**DOI:** 10.3390/diagnostics14060581

**Published:** 2024-03-08

**Authors:** Andreas Filis, Kay Engellandt, Sergio M. F. Romualdo, Ibrahim El-Battrawy, Dino Podlesek, Tareq A. Juratli, Ilker Y. Eyüpoglu, Mido Max Hijazi

**Affiliations:** 1 Department of Neurosurgery, Technische Universität Dresden, Faculty of Medicine, and University Hospital Carl Gustav Carus, Fetscherstrasse 74, 01307 Dresden, Germany; afilis@neuromaster.gr (A.F.); sergiomiguelfernandes.romualdo@ukdd.de (S.M.F.R.); dino.podlesek@ukdd.de (D.P.); tareq.juratli@ukdd.de (T.A.J.); ilker.eyuepoglu@ukdd.de (I.Y.E.); 2 Institute of Diagnostic and Interventional Neuroradiology, Technische Universität Dresden, Faculty of Medicine, and University Hospital Carl Gustav Carus, Fetscherstrasse 74, 01307 Dresden, Germany; kay.engellandt@ukdd.de; 3 Department of Cardiology, Bergmannsheil University Hospitals, Ruhr University Bochum, Bürkle de la Camp-Platz 1, 44789 Bochum, Germany; ibrahim.elbattrawy2006@gmail.com

**Keywords:** SDAVF, spinal arteriovenous fistula, myelopathy, endovascular treatment, surgical treatment, spinal cord edema, spinal angiography

## Abstract

Background: Successful treatment of spinal dural arteriovenous fistulas (SDAVF) requires prompt diagnosis with definitive fistula localization and non-delayed treatment. Magnetic resonance imaging (MRI) is used for the screening and follow-up of SDAVF, although the value of MRI signs such as myelopathy and flow voids is controversial. Therefore, we investigated the predictive value of MRI signs pre- and post-treatment and their correlation with the neurological status of SDAVF patients. Methods: We retrospectively analyzed the clinical records of 81 patients who underwent surgical or endovascular treatment for SDAVF at our hospital between 2002 and 2023. A total of 41 SDAVF patients with follow-up MRI of 4.6 [2.9–6.5] months (median [interquartile range]) post-treatment and clinical follow-up of 3, 6, and 12 months were included. Results: The extent of pretreatment myelopathy was seven [6–8] vertebral levels, with follow-up MRI showing no myelopathy in 70.7% of cases. The pretreatment flow voids extended over seven [4.5–10] vertebral levels and completely disappeared on follow-up MRI in 100% of cases. The modified Aminoff–Logue scale of disability (mALS) was four [2–7] pretreatment and two [0–4.5] at the third follow-up, with improvement in 65.9% of patients. The American Spinal Injury Association motor score (ASIA-MS) was 97 [88–100] pretreatment and 100 [95–100] at the third follow-up assessment, with 78% of patients improving. Pretreatment ASIA-MS correlated with the extent of myelopathy at admission (R^2^: 0.179; 95% CI: −0.185, −0.033; *p* = 0.006) but not with flow voids at admission, while pretreatment mALS showed no correlation with either MRI signs. The improvement in ASIA-MS and mALS between admission and the last follow-up showed no correlation with the extent of pretreatment myelopathy and flow voids or with pos-treatment MRI changes. The diagnostic sensitivity of magnetic resonance angiography (MRA) for localization of the fistula was 68.3% (28/41). Conclusions: The severity of the clinical condition in SDAVF patients has a multifactorial cause, whereby the ASIA-MS correlates with the extent of myelopathy pretreatment. MRI changes after treatment showed no correlation with the clinical outcome and cannot be used as a prognostic factor.

## 1. Introduction

Spinal dural arteriovenous fistula (SDAVF) results from a spinal anomalous connection between radiculomeningeal arteries and radicular veins, leading to venous hypertension and myelopathy [1,2,3,4,5,6,7]. Diagnosis is often delayed and made after misdiagnosis has led to unnecessary invasive procedures or inadequate treatment [5,6,8]. Magnetic resonance imaging (MRI) is used for screening and follow-up of SDAVF, although digital subtraction angiography (DSA) remains the gold-standard diagnostic procedure and is needed to identify the spinal location of the fistula and confirm the diagnosis [9,10]. Treatment consists of surgical or endovascular closure of the fistula, with surgery being the treatment of choice [11,12,13].

A combination of gait disturbances, lower limb weakness, back pain, sensory disturbances (paresthesia, hypesthesia, anesthesia, or hyperesthesia), and bowel and bladder dysfunction characterizes the clinical manifestations of SDAVF [10]. The onset of these symptoms is progressive, with a gradual deterioration over a period of 6 months to 2 years, although rapid deterioration has also been reported [14]. The aim of treatment is to interrupt the fistulous arterial and venous points [15,16,17,18,19].

Prompt diagnosis with definitive localization of the fistula and timely treatment are required for clinical improvement in SDAVF patients [20,21,22,23,24,25]. The association between MRI signs such as myelopathy and flow voids on pre- and post-treatment MRIs on the one hand and the clinical condition and outcome of the SDAVF patient on the other remains unclear [26]. Some previous studies have reported that the extent of myelopathy and flow voids on pretreatment MRI reflects the severity of neurological dysfunction at admission [27,28]. Other studies claim the opposite [29,30]. Few studies have addressed the value of post-treatment MRI for the clinical outcome of SDAVF [26]. In the present study, we used a retrospective design to evaluate the predictive value of pre- and post-treatment MRI signs in patients with SDAVF after successful treatment.

## 2. Materials and Methods

### 2.1. Study Design and Patient Data

#### 2.1.1. Study Design

**Figure 1:**
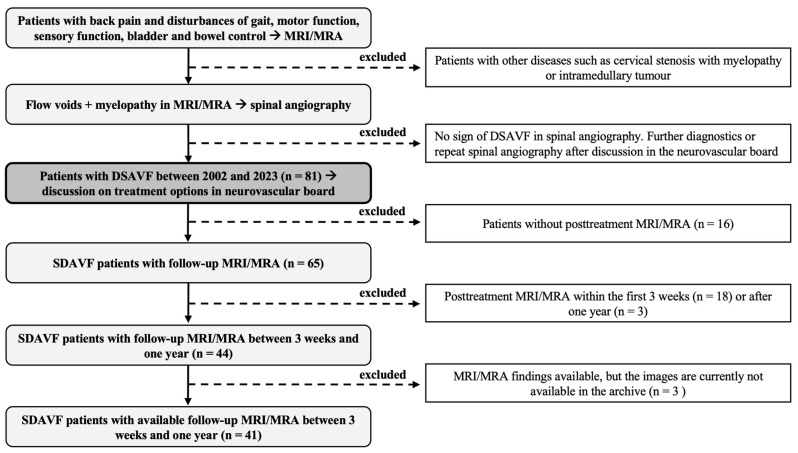
Study design: This figure shows our study design. MRI: magnetic resonance imaging; MRA: magnetic resonance angiography; DSA: digital subtraction angiography.

We conducted a retrospective analysis of patients with SDAVF who underwent surgery or endovascular treatment at our endovascular and spine center between 2002 and 2023. Patients with suspected SDAVF on MRI (vascular myelopathy and flow voids), corresponding symptoms (gait dysfunction, sensory disturbances, motor deficits, bowel and bladder dysfunction, or back pain), and evidence of SDAVF on spinal DSA were included in the study. A total of 81 patients with SDAVF were identified, of whom 65 underwent follow-up MRI after surgical or endovascular occlusion of SDAVF. Follow-up MRI was only performed in 44 patients between three weeks and one year, with 3 patients presenting documented findings, but the images were no longer available in the picture archiving and communication system (PACS). A total of 41 patients with a successfully treated SDAVF and available follow-up MRI images between three weeks and one year were included (Figure 1). Patients without evidence of SDAVF or follow-up MRI within the first three weeks or after one year were excluded.

#### 2.1.2. Patient Data and Institutional Review Board

The local ethics committee of the University Hospital Carl Gustav Carus in Dresden reviewed and approved our study (Ref: BO-EK-437102023). Patient data were collected via the ORBIS system (ORBIS, Dedalus, Bonn, Germany) and imaging examinations via the local PACS system (IMPAX, Impax Asset Management Group plc, London, UK). Spinal MRI, magnetic resonance angiography (MRA), and DSA were available in the IMPAX for review and assessment.

The following data were collected from the electronic medical records: age; gender; time from symptoms to MRI diagnosis; time from MRI diagnosis to surgery or embolization; history of comorbidities (vascular disease, coronary artery disease, stroke, hypertension, degenerative spine disease, bleeding medications, corticosteroid use and body mass index (BMI)); pre- or post-treatment MRI/MRA/DSA; number of incomplete or failed closures; number of secondary treatments performed (surgical or endovascular); complications related to treatment or hospitalization; side of fistula; location of fistula; first symptom; neurological status pretreatment, at the time of discharge, at first follow-up (3 months after treatment), at second follow-up (6 months after treatment), and at third follow-up (12 months after treatment); the American Spinal Injury Association motor score (ASIA-MS); and the modified Aminoff–Logue scale of disability (mALS).

### 2.2. Clinical Management

Clinical symptoms such as back pain and gait, sensory, motor, bowel, or bladder dysfunction, in conjunction with myelopathy and flow voids on MRI/MRA, are the basis for the diagnosis of SDAVF. Each case was discussed on a multidisciplinary board with neurointerventional radiologists and neurosurgeons, and the diagnosis was confirmed by spinal DSA. If two therapeutic options were considered, the patient was usually informed and educated about both treatment options, and the decision about the procedure was left to the patient. Endovascular treatment was preferred as a less invasive procedure in our hospital until around 2012. At that time, surgical treatment was suggested if endovascular treatment failed or was not feasible (vertebral artery or Adamkiewicz proximity with unintended risk of embolism). Since around 2012, surgical treatment has been the treatment of choice in our hospital, and endovascular therapy has been considered as an alternative.

In all cases, DSA and MRI/MRA were carefully reviewed by the neurosurgeon and a neurointerventional radiologist prior to treatment to determine the exact location and side of the fistula. Post-treatment spinal DSA and MRA/MRA were always performed after endovascular treatment. In most cases, a DSA and/or MRI/MRA were performed within the first three days after surgical treatment to assess fistula closure and any post-treatment complications. At 3, 6, and 12 months after surgical or endovascular treatment, an MRI/MRA was recommended to assess the disappearance of myelopathy and the regression of abnormal flow voids.

### 2.3. Illustrative Case

**Figure 2:**
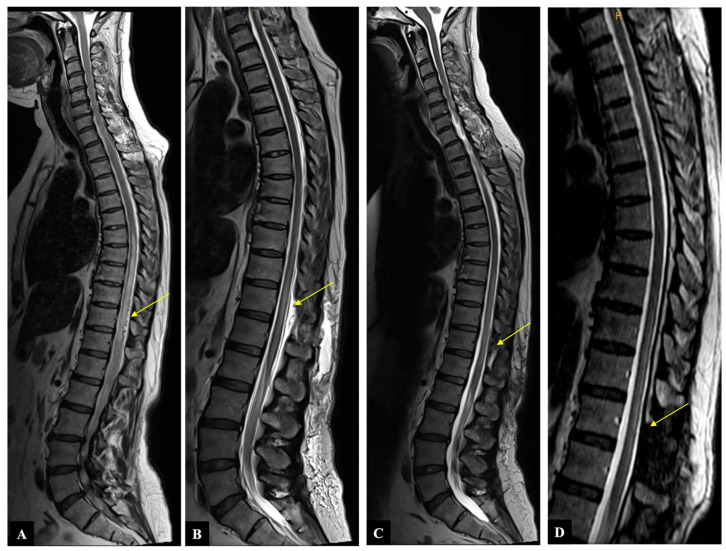
Case illustration: Sagittal T2-weighted MR image shows (**A**) preoperative spinal cord edema and flow voids (yellow arrow), (**B**) decrease in spinal cord edema and disappearance of flow voids immediately after operation (yellow arrow), (**C**) further decrease in spinal cord edema two months after operation (yellow arrow), (**D**) no myelopathy and no flow voids six months after operation (yellow arrow).

**Figure 3:**
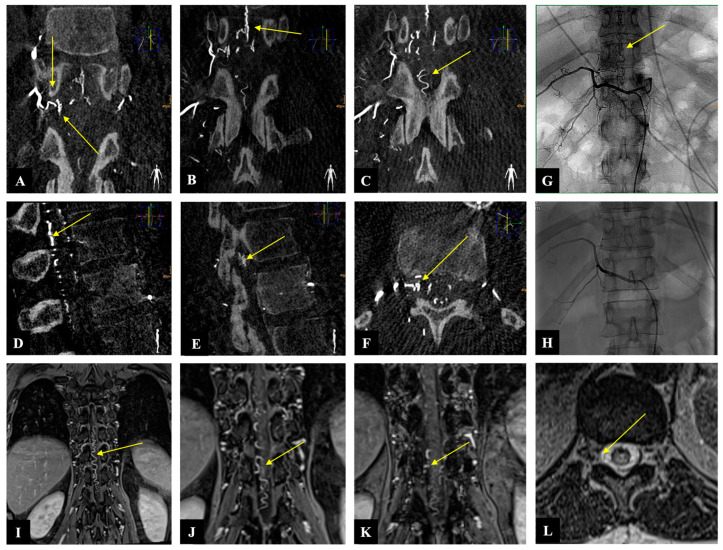
Case illustration: The images of three-dimensionally reconstructed digital subtraction angiography (DSA) of the spine ((**A**–**C**): coronary, (**D**,**E**): sagittal, and (**F**): axial) show the fistula location at the level of Th 12 on the right side as well as the flow voids (yellow arrows). Image (**G**) shows the preoperative SDAVF in conventional DSA with flow voids (yellow arrow), and image (**H**) demonstrates the postoperative absence of the SDAVF. The magnetic resonance angiography images ((**I**–**K**): coronary and (**L**): axial) show the fistulous point at the level of Th 12 on the right side as well as the flow voids (yellow arrow).

A 54-year-old woman presented with a one-month history of progressive gait disturbance, bowel and bladder dysfunction, and saddle anesthesia. The possible walking distance without a break was 200 m. There was also a slight paresis of the hip flexor and big toe extensor on the right side (ASIA-MS: 98, mALS: 6). The patient was admitted to the hospital by her general practitioner. On the day of admission, we performed a spinal MRI/MRA and DSA, which showed a DSAVF at the Th12/L1 level on the right. On the same day, the patient developed a rapidly progressing high-grade paraparesis with complete urinary and fecal incontinence (ASIA-MS: 70, mALS: 11). Emergency surgical treatment was performed via hemilaminectomy and closure of the right Th12 SDAVF without complications. Post-treatment DSA showed complete obliteration of the fistula, and MRI revealed no further flow voids and a reduction in myelopathy. The patient could be mobilized on the ward floor. After 2 months, the patient presented to the emergency room without motor deficits (ASIA-MS 100) but with a renewed deterioration of gait (gait score in mALS: 2) and persistent saddle anesthesia and leg paresthesia. The MRI showed further regression of the myelopathy and no signs of SDAVF recurrence. The gait disturbance improved spontaneously, and the patient was discharged home. At the second follow-up assessment (after 6 months), the MRI showed no myelopathy, but the patient still had a gait disturbance. A return to work was no longer possible. This case is the only one from our center with a rapid deterioration within one day, which we do not know in this form for this disease (Figure 2 and Figure 3).

### 2.4. Statistical Analysis

Statistical analysis of the data was performed using the SPSS software package (SPSS Statistics 29, IBM, Armonk, New York, NY, USA). Descriptive statistics were used, and categorical variables were tested by Fisher exact tests or chi-square tests. Numerical variables were analyzed with Mann–Whitney U tests. All statistical tests were two-sided, and a *p* value < 0.05 was considered statistically significant.

A linear regression analysis was performed to evaluate the correlation between the extent of myelopathy and flow voids before treatment on the one hand and pretreatment mALS and ASIA-MS, post-treatment mALS and ASIA-MS, and improvement in mALS and ASIA-MS on the other hand. In addition, a binary logistic regression analysis was performed to determine the correlation between the improvement in myelopathy and flow voids at follow-up MRI and the improvement in mALS and ASIA-MS at the last follow-up.

## 3. Results

### 3.1. Patient Characteristics

Our study population consisted of 11 women (26.8%) and 30 men (73.2%) with an age of 65.9 [54.5–73.5] years (median [interquartile range]). The duration from symptoms to MRI diagnosis was 7 [2.5–24] months, and the time from suspected MRI diagnosis to surgical or endovascular treatment was 15 [9–34.5] days (Table 1).

The extent of myelopathy at admission was seven [6–8] vertebral levels, and in 70.7% of patients (*n*: 29), there was no evidence of myelopathy on follow-up MRI. The interval between treatment and follow-up MRI was 4.6 [2.9–6.5] months. The flow voids at admission were extended along seven [4.5–10] vertebral levels and were 100% completely absent on follow-up MRI (median 4.6 months).

The mALS score at admission was 4 [2–7] and 2 [0–4.5] at the third follow-up, with an improvement in mALS between admission and the last follow-up observed in 65.9% of patients (*n*: 27). The ASIA-MS at admission was 97 [88–100] and 100 [95–100] at the third follow-up, with an improvement in ASIA-MS between admission and the last follow-up seen in 32 patients (78%).

The majority of fistulas were located in the lower thoracic (17, 41.5%), followed by the lumbar (12, 29.2%), upper thoracic (7, 17.1%), cervical (3, 7.3%), and sacral (2, 4.9%). There were 18 left fistulas, 22 right fistulas, and 1 bilateral fistula. Two patients (4.9%) had an initial incomplete or failed occlusion, which was then occluded definitively with a second procedure.

Treatment- or hospital-related complications were reported in 14.6% of patients (*n*: 6). The diagnostic sensitivity of MRA to locate the fistula was 68.3% (*n*: 28). Surgery was performed in 36 patients (87.8%) and embolization in 5 patients (12.2%). The BMI was 27.5 [24.9–30.3] kg/m^2^.

### 3.2. Myelopathy and Flow Voids

We used simple linear regression analysis to identify the correlation between the extent of myelopathy at admission on the one hand and clinical symptoms before treatment and one year after treatment using ASIA-MS and mALS on the other. A correlation was only observed between ASIA-MS and the extent of myelopathy (R2: 0.179; 95% CI: −0.185, −0.033; *p* = 0.006, Table 2). The improvement in ASIA-MS and mALS between admission and the last follow-up showed no correlation with the extent of myelopathy. The extent of flow voids showed no correlation to the clinical features or outcomes of SDAVF.

### 3.3. Improvement in mALS and ASIA-MS

Using binary logistic regression, no correlation was found between the absence of myelopathy in the follow-up MRI and the improvement in mALS or ASIA-MS in the last follow-up (Table 3). The analysis of a correlation between flow voids on follow-up MRI and the improvement in mALS or ASIA-MS in the last follow-up could not be performed as all patients had no flow voids in the follow-up MRI.

## 4. Discussion

The main finding of our study and from our more than 20 years of monocentric experience with SDAVF showed that the extent of myelopathy on MRI at admission was related to patients’ motor deficits but not to overall clinical conditions, such as gait, urination, and defecation function. In contrast, follow-up MRI changes showed no correlation with clinical outcome and cannot be used as a prognostic factor.

Our study population was predominantly men (three men to one woman) in the six decades of life, as reported in previous studies [10]. In our cohort, the diagnosis of SDAVF was delayed by a median of 7 months, and it took a median of 15 days before endovascular or surgical treatment was performed. The interval between treatment and follow-up MRI was 4.6 months. MRI was performed at the first follow-up (3 months) in some patients and at the second follow-up (6 months) in others, so there was no consistent performance of MRI for evaluation. Patients who received an MRI during their stay or after one year were excluded from the study due to standardization.

The extent of myelopathy on MRI at admission in our cohort was seven vertebral levels long. Shin et al. reported a 5.2 vertebral level average length of myelopathy in their cohort of 15 patients [9]. Luo et al. considered the length of myelopathy over five vertebral bodies as an indicator of severe neurological dysfunction and found a correlation with clinical outcome [26]. From our point of view, it is difficult to determine at what length of myelopathy severe neurological deficits are to be expected. Various factors are involved, such as the localization of the myelopathy in the spinal cord (cervical and conus) and the degree of spinal stenosis due to the flow voids. The absence of myelopathy on follow-up MRI was found in 70% of our patients, as in an earlier study (73%, 4/15 patients) [9]. The extent of flow voids in our study was similar to myelopathy and was seven vertebral levels long, supporting the pathophysiology of myelopathy based on venous hypertension. No flow voids were seen on follow-up MRIs in all patients. If these persist, incomplete or failed fistula closure is conceivable.

Our cohort has shown that almost half of SDAVFs are localized in the lower thoracic spine, and there is no side preference, although some studies claim that the pathology occurs predominantly on the left side [31,32]. The diagnostic sensitivity of MRA for the fistula location has not yet been investigated. We compared the results of the MRA examination before DSA with the DSA result for the fistula location and found a diagnostic sensitivity of 68.3% (28/41) for the fistula location and side. This is an unsatisfactory result overall but offers good sensitivity for a non-invasive and radiation-free examination and should be used to reduce the radiation exposure of angiography beforehand.

In our cohort, the mALS was 4 at admission and improved to 2 at the third follow-up. Overall, 65.9% of patients had an improvement in mALS between admission and the last follow-up. The mALS values are consistent with previous studies and showed the main improvement between admission and hospital discharge [6,9,26]. In course, even after one year, there was only a slight improvement in the mALS values. The median ASIA-MS was 97 at admission and 100 at the third follow-up, with overall improvement between admission and the last follow-up in 78% of patients. To our knowledge, the use of ASIA-MS in SDAVF has not yet been performed. The reason we have used this detailed score is because mALS does not provide conclusive information about the motor deficits that we have observed more frequently in severe myelopathy. The regression analysis in our study showed a significant correlation between ASIA-MS and the extent of myelopathy on MRI at admission, confirming our clinical hypothesis.

In our study, we could not confirm the discussed correlation of MRI signs (myelopathy and flow voids) with mALS at admission and the correlation of their change on follow-up MRI with the clinical outcome. Therefore, we agree with the prevailing opinion that the change in the follow-up MRI does not allow any conclusions to be drawn about the clinical condition of the patients.

A post-treatment DSA is the method of choice to confirm the closure of the fistula. An MRI examination remains optional as long as the patient shows clinical improvement. The flow voids usually disappear after approximately one week. A reduction in myelopathy can be observed after one week, and a complete disappearance can be seen after three months, which shows no clinical correlation. We recommend postoperative DSA and MRI examinations in 3 months. If the clinical symptoms worsen or persist, we recommend prompt MRI control.

### Limitations and Strengths of This Study

The monocentric, retrospective nature of our analysis, the long inclusion interval, and the limited number of SDAVF patients (41 patients) might reduce the external validity of our study. Furthermore, our analysis could be affected by a possible selection bias due to our treatment flow charts, as our experience has been to favor surgery over embolization. Nevertheless, our cohort analysis is based on a 20-year treatment period of SDAVF in a large university neurosurgery center, suggesting a high internal validity of our study. The clinical follow-up and the follow-up MRI examination were not performed at the same time. Therefore, our observations may be useful to understand the clinical and radiologic characteristics of SDAVF.

## 5. Conclusions

SDAVF is a rare but well-treatable disease in which mALS and ASIA-MS improve over the disease course. Although MRI forms the basis for the diagnosis of SDAVF, it does not provide sufficient diagnostic sensitivity for the localization of the fistula. It offers a good non-invasive and radiation-free option for narrowing down the potential localization in advance and should be used to reduce the radiation exposure of angiography.

The severity of the clinical condition of SDAVF patients has a multifactorial reason, whereby the motor deficits correlate with the extent of the myelopathy. MRI changes at follow-up showed no correlation with the clinical outcome and cannot be used as a prognostic factor.

## Figures and Tables

**Table 1:** Baseline characteristics.

Variable	Value
Age, median [IQR]	65.9 [54.5–73.5] y
Female gender, *n* (%)	11 (26.8%)
Time from symptom to MRI diagnosis, median [IQR]	7 [2.5–24] m
Time from MRI diagnosis to treatment, median [IQR]	15 [9–34.5] d
Myelopathy extension at admission on MRI, median [IQR]	7 [6–8] v
Absence of myelopathy on FU MRI, *n* (%)	29 (70.7%)
Flow void extension at admission on MRI, median [IQR]	7 [4.5–10] v
Absence of flow voids on FU MRI, *n* (%)	41 (100%)
mALS at admission, median [IQR]	4 [2–7]
mALS at third FU, median [IQR]	2 [0–4.5]
ASIA-MS at admission, median [IQR]	97 [88–100]
ASIA-MS at third FU, median [IQR]	100 [95–100]
Interval between treatment and FU MRI, median [IQR]	4.6 [2.9–6.5] m
Fistulous point:	
Cervical, *n* (%)	3 (7.3%)
Upper thoracic, *n* (%)	7 (17.1%)
Lower thoracic, *n* (%)	17 (41.5%)
Lumbar, *n* (%)	12 (29.2%)
Sacral, *n* (%)	2 (4.9%)
Side of fistula, *n*	R: 22, L: 18, B: 1
Incomplete or failed occlusion, *n* (%)	2 (4.9%)
Treatment- or hospital-related complications, *n* (%)	6 (14.6%)
Improvement in mALS between admission and last FU, *n* (%)	27 (65.9%)
Improvement in ASIA-MS between admission and last FU, *n* (%)	32 (78%)
Diagnostic sensitivity of MRA to locate the fistula, *n* (%)	28 (68.3%)
Surgery vs. embolization, *n* (%)	36 (87.8%) vs. 5 (12.2%)
BMI	27.5 [24.9–30.3] kg/m^2^

IQR: interquartile range; MRI: magnetic resonance imaging; MRA: magnetic resonance angiography; *n*: number; y: year; m: month; d: day; v: vertebral body; mALS: modified Aminoff–Logue scale of disability; ASIA-MS: American Spinal Injury Association motor score; FU: follow-up; BMI: body mass index; R: right; L: left; B: both.

**Table 2:** Correlation between MRI signs and clinical features and outcomes of SDAVF patients.

Associated Variable	Simple Linear Regression					
	B	(95% CI)	SE	β	R^2^	*p*
**Extent of pre. myelopathy** Pre. ASIA-MS	−0.109	−0.185, −0.033	0.037	−0.423	0.179	**0.006**
Third FU ASIA-MS	−0.058	−0.187, 0.071	0.062	−0.212	0.045	0.357
Improved ASIA-MS	0.410	−2.072, 2.892	1.227	0.053	0.003	0.740
Pre. mALS	0.319	−0.002, 0.641	0.159	0.306	0.094	0.052
Third FU mALS	0.331	−0.172, 0.834	0.240	0.302	0.091	0.184
Improved mALS	−0.503	−2.666, 1.661	1.070	−0.075	0.006	0.641
**Extent of pre. Flow voids** Pre. ASIA-MS	−0.075	−0.168, 0.018	0.046	−0.251	0.063	0.113
Third FU ASIA-MS	−0.003	−0.140, 0.134	0.065	−0.012	0.000	0.960
Improved ASIA-MS	−0.375	−3.229, 2.479	1.411	−0.430	0.002	0.792
Pre. mALS	0.195	−0.189, 0.578	0.189	0.162	0.026	0.311
Third FU mALS	0.117	−0.429, 0.662	0.261	0.102	0.010	0.660
Improved mALS	−0.011	−2.504, 2.483	1.233	−0.001	0.000	0.993

B: unstandardized coefficient, CI: confidence interval; SE: standard error; β: standardized coefficient; R^2^: coefficient of determination; Pre.: pretreatment; mALS: modified Aminoff–Logue scale of disability; ASIA-MS: American Spinal Injury Association motor score; FU: follow-up. Bold values are significant results (*p* < 0.05) as indicated in the methods.

**Table 3:** Correlation between the absence of myelopathy on MRI and clinical improvement.

Associated Variable	Binary Logistic Regression	
	OR (95% CI)	*p* Value
**Absence of myelopathy on FU MRI**		
Improved mALS in the last FU	0.630 (0.157–2.533)	0.515
Improved ASIA-MS in the last FU	4.190 (0.463–37.938)	0.202

OR: odds ratio; CI: confidence interval; MRI: magnetic resonance imaging; mALS: modified Aminoff–Logue scale of disability; ASIA-MS: American Spinal Injury Association motor score; FU: follow-up.

## Data Availability

The original contributions presented in this study are included in the article. Further inquiries can be directed to the corresponding author.
